# Contribution of the *LAC* Genes in Fruit Quality Attributes of the Fruit-Bearing Plants: A Comprehensive Review

**DOI:** 10.3390/ijms242115768

**Published:** 2023-10-30

**Authors:** Muhammad Khalil Ullah Khan, Xiaojie Zhang, Zitan Ma, Mingxia Huang, Ce Yang, Xiaoming Wang, Mengjun Liu, Jianying Peng

**Affiliations:** 1 College of Horticulture, Hebei Agricultural University, Baoding 071001, China; grankhan797@gmail.com (M.K.U.K.); zhangxiaojietyy@163.com (X.Z.); mazitan2023@163.com (Z.M.); hmx15886915901@163.com (M.H.); 15530416500@163.com (C.Y.); wxm19980528@163.com (X.W.); 2 Research Center of Chinese jujube, Hebei Agricultural University, Baoding 071001, China

**Keywords:** *LAC* genes, endocarp, flesh lignification, defense, pigmentation, degradation, synthesis

## Abstract

Laccase genes produce laccase enzymes that play a crucial role in the production of lignin and oxidation reactions within plants. Lignin is a complex polymer that provides structure and toughness to the cell walls of numerous fruit plants. The *LAC* genes that encode laccase enzymes play vital roles in plant physiology, including the synthesis of pigments like PA that contribute to the colors of fruits, and in defending against pathogens and environmental stresses. They are crucial for fruit development, ripening, structural maintenance in plants, and adaptation to various environmental factors. As such, these genes and enzymes are essential for plant growth and development, as well as for various biotechnological applications in environmental remediation and industrial processes. This review article emphasizes the significance of genes encoding laccase enzymes during fruit growth, specifically pertaining to the strengthening of the endocarp through lignification. This process is crucial for ensuring fruit defense and optimizing seed scattering. The information gathered in this article will aid breeders in producing future fruit-bearing plants that are resistant to disease, cost-effective, and nutrient-rich.

## 1. Introduction

The *LAC* (Laccase) genes encode an enzyme called laccase, which is involved in lignin biosynthesis and oxidative reactions in plants [1,2]. Lignin is a complex polymer that makes up the cell walls of many plants and is responsible for their strength and rigidity [1,3]. In fruit-bearing plants, the *LAC* genes play a crucial role in the development and ripening of fruit [3]. Laccase enzymes are involved in the synthesis of lignin in the cell walls of fruits, which contributes to their firmness and texture. The activity of *LAC* genes decreases during the fruit ripening process, leading to softening and the release of aroma and flavor compounds.

Additionally, laccase enzymes are involved in the biosynthesis of pigments such as proanthocyanidins (PAs), which give fruits their characteristic colors [4]. *LAC* genes are also involved in the defense mechanisms of plants against pathogens and environmental stresses [5]. Hence, the *LAC* genes are essential for the growth, development, and ripening of fruits in plants, as well as for their structural integrity and response to environmental factors [5,6]. Laccases are enzymes that play a key role in lignin biosynthesis, which is the process by which plants make cell walls and provide them with structural support. In the endocarp of fruits, lignification can play a role in protecting seeds from environmental stresses and preventing premature germination. LAC enzymes catalyze the oxidative polymerization of monolignols, which are precursors to lignin, and contribute to the formation of the lignin polymer network in the endocarp cells [3]. Therefore, *LAC* is an essential component in the endocarp lignification process.

This review article describes the comprehensive role of the *LAC* gene family in fruit-bearing plants. Further, the *LAC* gene family, which encodes for laccase enzymes, is known to play a critical role in lignin biosynthesis and plant development. The study highlights the importance of laccase enzymes in fruit development, particularly in the lignification of endocarp, which is critical in providing fruit protection and enhancing seed dispersal.

## 2. Molecular Regulation of the *LAC* Gene Family Involved in Fruit Lignification

It is well known that lignification is the process by which plants produce lignin, a complex polymer that provides structural support and protection to the plant cell wall. In fruits, lignification is involved in the development of the fruit’s texture and resistance to mechanical stress and pathogens [3]. The *LAC* gene family, which encodes laccase enzymes, is known to be involved in lignin biosynthesis in plants [2]. Lignin biosynthesis begins with the synthesis of phenylpropanoids, which are then polymerized into lignin [2]. One of the key steps in this process is the oxidation of monolignols, which are the monomeric building blocks of lignin. Laccase enzymes catalyze this oxidation step, and the *LAC* gene family encodes laccases in plants [1]. Furthermore, laccases (*LAC*s) are a class of oxidases in plants that possess three conserved blue copper protein domains and have been linked to lignin polymerization in *Populus trichocarpa* and Arabidopsis [7,8]. In *P. trichocarpa*, five *LAC* genes (*LAC1*, *LAC2*, *LAC3*, *LAC90*, and *LAC110*) have been identified based on their expression during xylem lignification, and previous research has utilized RNA interference to suppress these genes, resulting in a decrease in both secondary cell wall thickness and lignin content in xylem fibers of transgenic Populus plants [8,9].

Also, in fruits, the expression of *LAC* genes is regulated by hormonal and developmental cues. For example, in apple fruit, the expression of *LAC* genes is upregulated during the late stages of fruit development, which coincides with lignin deposition [10]. This upregulation is correlated with an increase in the activity of the transcription factors activated by PbMYB26, which directly bind to the promoters of the *PbLAC4-like* gene and activate its expression [11]. In addition to transcriptional regulation, *LAC* gene expression is also post-transcriptionally regulated by microRNAs (miRNAs) [5]. MiRNAs are short RNA molecules that can bind to complementary sequences in mRNA and inhibit their translation or promote mRNA degradation.

In more detail, the regulation of the *LAC* gene family is complex and involves multiple levels of regulatory control. However, understanding the molecular mechanisms involved in lignification and the regulation of *LAC* genes is important for improving fruit quality and resistance to environmental stresses [12,13]. Future research in this field will continue to shed light on the molecular mechanisms involved in fruit lignification and the potential applications of this knowledge in fruit breeding and biotechnology.

The members of the *LAC* gene family have been found to be involved in both endocarp and flesh lignification. The detail about the role of *LAC* genes in fruit lignification is as follows.

### 2.1. Molecular Mechanism of LAC Genes Involved in Fruit Endocarp Lignification

The hardening of endocarp in drupes and dehiscent fruits during maturation is characterized by the formation of a secondary cell wall and lignification. Although the formation process of secondary walls in fruit tissues is not well understood, information on this can be extrapolated from studies on wood formation [3,14]. In woody tissue, the transformation of fleshy xylem cells into woody tissue involves elongation, expansion, secondary cell wall deposition, programmed cell death, and heartwood formation [3,14]. Secondary walls are composed of multiple cellulose, hemicellulose, and lignin layers, along with smaller amounts of proteins and pectin [3,14].

Lignin is a complex compound that typically contains p-hydroxyphenyl (H), guaiacyl (G), and syringyl (S) units [15]. These units are formed by the polymerization of the monolignols *p*-coumaryl alcohol, coniferyl alcohol, and sinapyl alcohol, respectively [15,16]. Monolignol glucosides are the dominant form of monolignols in the cytoplasm due to their low water solubility and potential toxicity.

Fruits contain relatively low levels of lignin compared to other plant tissues such as wood and stems. However, the lignin composition in fruit varies depending on the type of fruit and its maturity stage. In some fruits, such as cherries, lignin content decreases during the ripening process [1,3,14]. Cherries contain mainly G lignin, with very low levels of S units. Similarly, grape skins have been found to contain mainly G lignin, with some H units. On the other hand, some fruits such as avocado and olive contain relatively high levels of lignin in their seeds. Avocado seeds have been found to contain mainly S lignin, while olive seeds have been reported to contain a mixture of G, S, and H units. While lignin is not considered a major component of fruits, its presence in certain parts of fruits such as seeds can impact their potential uses in industrial applications such as biofuel production [1].

Extensive research has been conducted on lignin biosynthesis pathways, and the enzymes involved have been extensively described [17,18]. Cytochrome P450 oxidoreductases, including (C4H), (C3H), and (F5H), are responsible for aromatic ring hydroxylation [15], while other monolignol biosynthetic enzymes, such as (PAL), (4CL), (HCT), (CSE), (CcOAMT), (CCR), (COMT), and (CAD), are present in the cytoplasm [19].

During glycoside formation, monolignols must be transported to the secondary cell wall via (UGT) and β-glucosidase (BGLU), which are then oxidized and polymerized into lignin by (POD) and (LAC) [19]. C3H and HCT are critical enzymes that regulate the contents of H-monolignol and G/S-monolignin [15]. The lignification processes in peach and Arabidopsis share common regulators, while the regulation of lignification in apricots may involve structural genes such as *CAD*, *POD*, and *LAC*, according to transcriptome analysis [20]. The transcription factor NST1 can also regulate *CAD* and thus play a role in lignin biosynthesis [21].

Lignification exhibits significant flexibility with other monomer units, including conferyl esters, coumarates, flavone tricin, dilignols, and trilignols. In dicotyledonous plants, such as pear trees, the lignin found in xylem tissues is primarily made up of G and S units, with few H types [15,18]. During the lignification process in pear stone cells, several compounds like p-coumaric acid, ferulic acid, sinapaldehyde, coniferyl alcohol, and sinapyl alcohol were observed. Regulating the intermediate metabolite contents, particularly p-coumaric acid, can enhance the pear’s quality [22].

Lignin plays a vital role in providing a framework within secondary cell walls for the polymerization of cellulose and hemicellulose polymers, which together contribute to tissue rigidity and tensile strength [14]. The major enzymes involved in the lignin pathway and potential regulatory points have been identified, and lignin is formed from the phenylpropanoid pathway, resulting in coniferyl and sinapyl alcohols [14]. The process of lignification involves the oxidative reaction of these monomers, assisted by peroxidases and laccases [14]. The multistep radical coupling of the monomers, particularly the cross-coupling with the growing polymer, produces the complex lignin polymer [3,14]. The role of the *LAC* genes in endocarp lignification fruit is as follows.

Similarly, in their study, Zhang et al. [19] examined the metabolite dynamics and genetic regulatory mechanisms of apricot endocarp during and after lignification. They utilized ultra-performance liquid chromatography–tandem mass spectrometry to analyze the endocarp of the kernel-using apricot cultivar ‘Youyi’ [19]. The researchers observed a rapid increase in endocarp thickness from 8 to 37 days after flowering (DAF), followed by the deposition of lignin at 37 DAF. They identified 626 non-volatile metabolites in the endocarp tissues before (33 DAF) and after (41 and 45 DAF) the onset of lignification. As lignification progressed, the levels of sugar and organic acid decreased, while L-phenylalanine and L-tyrosine increased. Prior to lignification, phenylpropanoid metabolites such as p-coumaric acid, ferulic acid, neochlorogenic acid, dicumarol, conifer-in, and some lignans were present [19]. However, after lignification, glycoside lignin or lactone coumarins were the main metabolites with increased relative amounts of L-asarinin and forsythin. The genes responsible for lignin biosynthesis, such as β-glucosidase, coniferyl-alcohol glucosyltransferase, and laccases, were upregulated and played a key role in the acceleration of lignification.

**Figure 1:**
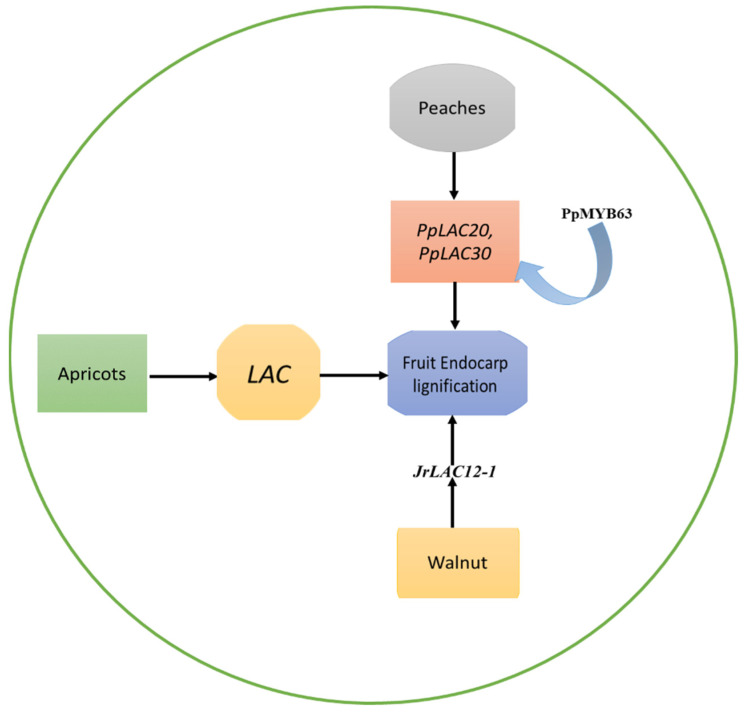
The role of the *LAC* genes in endocarp lignification of apricots, peaches, and walnuts. The *LAC*s play a role in the formation of lignified endocarp in the kernel of apricots. *PpLAC20* and *PpLAC30* were identified as the most important members involved in the biosynthesis of peach lignin. A peach MYB transcription factor PpMYB63, which is similar to AtMYB58 and AtMYB63, was found to have the ability to activate the promoters of *PpLAC20* and *PpLAC30.* Similarly, the development of the walnut endocarp’s lignification process is notably impacted by laccase genes, with *JrLAC*s having specific functions in the fruit’s developmental stages.

This research offers a new understanding regarding the development of lignified endocarp in apricots that utilize the kernel, as well as the functions of LACs and other enzymes, in monolignin transport and oxidative polymerization [19], as shown in Figure 1.

Moreover, studies on the mechanisms of endocarp hardening in peaches have been limited, with most examining only one or two components of enzymes involved in the composition and formation of the stone tissue [3,23]. However, Ryugo’s work in the 1960s documented that peach stones are rich in lignin, which accumulates seasonally, and identified the presence of intermediates in lignin biosynthesis [24,25]. Further studies have shown that the dry weight of the stone and lignification increase from the second stage of fruit development until maturity [23].

More recent research has analyzed the biochemistry of drupes, including peaches, coconuts, black walnuts, and olives, revealing that they contain almost twice as much lignin as wood [26]. This finding suggests that the process of secondary wall formation can occur to a significant extent in fruit endocarp tissues. Therefore, a closer examination of the mechanisms underlying the formation of the secondary cell wall and lignification in endocarp tissues can lead to a better understanding of the process of endocarp hardening in peaches and other fruits [23].

Peach, scientifically known as *Prunus persica*, is a fruit tree of great economic significance that produces drupe fruits with hard stones encasing the seeds [27]. Laccases are copper-containing glycoproteins that play a crucial role in cell elongation and are present in peach plant genomes [26,27]. These enzymes are responsible for the polymerization of lignin, a necessary process that is vital for the hardening of the endocarp or stone, with laccase being the primary enzyme involved in the polymerization of monolignol [27].

In the peach genome, Qui et al. [27] discovered a total of 48 laccase genes (*PpLAC*s), which were unevenly spread out across eight chromosomes, and identified that they belong to six distinct phylogenetic groups (I-VI). Out of these, 15 *PpLAC*s were identified as potential targets of miR397, a critical inhibitor of lignin biosynthesis. Following the phylogenetic sequence, and spatiotemporal expression profile analysis, it was concluded that *PpLAC7*, *19*, *20*, *21*, *27*, *28*, and *30* are likely to contribute to lignin synthesis and endocarp hardness in peach fruit. Amongst these seven, *PpLAC*s, *PpLAC20,* and *PpLAC30* were identified as the most important members involved in the biosynthesis of peach lignin, based on their gene expression patterns [27]. Furthermore, a peach MYB transcription factor PpMYB63, which is similar to AtMYB58 and AtMYB63, was found to have the ability to activate the promoters of *PpLAC20* and *PpLAC30* [27], as shown in Figure 1.

Similarly, seed hardness is closely associated with the lignin biosynthesis pathway, where laccase plays a crucial role as the key enzyme in this process [2]. The presence of the RY-element was solely detected in the promotor of *PgLAC16*, indicating that the increased expression of *PgLAC16* may impede the accumulation of storage material during seed development, leading to the prevention of seed hardness in soft-seed pomegranate [2]. Hence, the significant expression of *PgLAC1*/*4*/*6*/*7*/*16* could be crucial in investigating the soft-seed formation and identifying candidate genes for breeding soft-seed pomegranate using GA application [2]. *PgLAC1*, *PgLAC6*, *PgLAC7*, and *PgLAC16* were found in higher quantities in the soft-seed pomegranate cultivar, while the hard-seed pomegranate cultivar exhibited more significant amounts of *PgLAC37* and *PgLAC50* [2].

Considered a significant crop across the globe, walnuts are highly valued for their nutritional content, comprising proteins, unsaturated fatty acids, and vitamins [28]. The shells surrounding walnut kernels play a crucial role in protecting the seeds from environmental stress. Lignin is the dominant chemical component of walnut shells, providing strength and stiffness. Walnuts, along with other drupe crops such as almonds, peaches, and pomegranates, develop hardened endocarps which grow from the inner layer of the ovary [29]. The formation of parenchymal cells, deposition of secondary cell walls, and differentiation into polylobate stone cells are key steps in shell development [29]. However, there is a limited understanding of the molecular mechanisms driving stone cell formation and endocarp hardening. Further research is necessary to uncover the genes and processes behind shell thickness, which has direct commercial implications for yield and kernel quality [29].

The lignification of the walnut endocarp is significantly influenced by laccase genes, and *JrLAC*s exhibit distinct roles in the developmental process of the fruit. The study suggests that *JrLAC12-1* likely plays a crucial role in the lignification of the endocarp [30], as shown in Figure 1. During the lignification stage, *JrLAC12-1*, *JrLAC12-2*, and *JrLAC16* experienced significant changes in gene expression [30]. When analyzing the expression of *JrLAC*s across various endocarp developmental stages and tissues, it became apparent that most *JrLAC*s were highly expressed in young tissues and had lower expression in mature tissues [30]. Additionally, *JrLAC12-1* exhibited particularly high expression levels in young stems. A strong positive correlation was observed between the expression of *JrLAC12-1* and the variations in endocarp lignin content [30]. The role of the important *LAC* genes in fruit endocarp lignification is shown in Table 1.

### 2.2. Molecular Mechanism of LAC Genes Involved in Fruit Flesh Lignification

The *LAC* genes, or laccase genes, encode for enzymes called laccases that are involved in a variety of biological processes such as lignin degradation, pigment biosynthesis, and defense against pathogens [31]. In fruit, laccases have been found to play a role in fruit ripening and browning [36]. One study conducted on postharvest strawberry fruit found that the expression of *LAC* genes was positively correlated with fruit ripening and softening [2,36]. The study also found that the activity of laccase enzymes increased during ripening, which may contribute to the breakdown of cell walls and the softening of fruit [10]. Another study on postharvest peach fruit found that the expression of *LAC* genes was upregulated during fruit browning, caused by mechanical damage. The study suggested that laccases were involved in the oxidation of phenolic compounds, which resulted in the brown coloration of the fruit [36]. Furthermore, a study on postharvest kiwifruit found that the expression of *LAC* genes was correlated with the production of lignin, which contributes to the firmness of the fruit. The study suggested that laccases may be involved in the regulation of lignin biosynthesis in kiwifruit [32]. Thus, the *LAC* genes play a role in fruit ripening, browning, and firmness by encoding laccase enzymes that are involved in the breakdown of cell walls, oxidation of phenolic compounds, and regulation of lignin biosynthesis. The potential role of the *LAC* gene in the flesh lignification of fruit is discussed as follows.

The researchers found that miR397a, a microRNA in Chinese pear, regulates lignification by suppressing the expression of *LAC* genes, which encode enzymes involved in lignin biosynthesis. Overexpression of *PbrmiR397a* and simultaneous silencing of three *LAC* genes led to reduced lignin content and stone cell number in pear fruit. These findings suggest a potential strategy for improving fruit quality through genetic modification [8]. The findings of this study suggest that miR397a and *LAC* genes play a crucial role in regulating lignification in fruit stone cells and that manipulating its expression can be a potential strategy to reduce the negative impact of stone cells on fruit quality [8]. The study results showed that the transgenic plants with reduced miR397a expression and silenced *LAC* genes had fewer vessel elements and thinner secondary walls in comparison to the wild-type control plants, resulting in decreased lignin content and stone cell numbers in pear fruit [8].

Furthermore, *PbMC1a*/*1b* and *PbRD21* were found to have a significant impact on the expression of genes and lignin levels in pear fruits and flesh calli when expressed simultaneously [37]. This suggests that *PbMC1a/1b* plays a crucial role in the lignification of cell walls, potentially by collaborating with *PbRD21* to enhance the mRNA levels of genes associated with lignin synthesis and facilitate the development of stone cells in pear fruit [37]. In addition to miR397a, the MYB transcription factor and the *LAC* genes also play important roles in fruit lignification. The MYB transcription factor is known to regulate lignin biosynthesis by binding to the promoter regions of *LAC* genes and activating their expression [31]. In fruit, MYB transcription factors have been found to regulate stone cell formation and lignification in apples and pears [31]. *LAC* genes encode for enzymes involved in lignin biosynthesis, including peroxidases and laccases. Knockdown or silencing of *LAC* genes has been shown to reduce lignin content and stone cells in fruit [31]. However, complete inhibition of these genes can also lead to negative effects on fruit development and quality [31]. Therefore, fine-tuning *LAC* gene expression through regulatory factors such as miRNAs and MYB transcription factors may provide a more precise approach to controlling fruit lignification and stone cell formation [31].

*PbrMYB169* is believed to function as a transcriptional activator, promoting the production of lignin and regulating the development of secondary walls in cells found in fruit stones [31]. PbrMYB169 protein was found to activate the promoters of several lignin genes including *C3H1*, *CCR1*, *CCOMT2*, *CAD*, *4CL1*, *4CL2*, *HCT2*, and *LAC18* through binding with AC elements [ACC(T/A)ACC] [31]. The overexpression of *PbrMYB169* in transgenic Arabidopsis plants led to an increase in the expression of lignin genes including *LAC18* and resulted in thicker cell walls and increased lignin deposition in vessel elements. The ratio of syringyl and guaiacyl lignin monomers, however, remained unchanged [31]. The role of some important *LAC* genes in fruit flesh lignification is shown in Table 1.

## 3. Molecular Regulation of *LAC* Gene Family Involved in Anthocyanin and PA Degradation/Biosynthesis in Fruit

There is limited evidence indicating that the *LAC* gene plays a role in the degradation of anthocyanin. This degradation has been observed to cause browning of the pericarp of different fruits. While numerous regulators of *LAC* have been discovered, they have all been linked to the accumulation of lignin rather than anthocyanin degradation. The laccase-like multicopper oxidase gene family of sweet cherry is involved in anthocyanin stability, which is important for pericarp browning and the retention of the appealing red color, as well as developmental events such as rocky pit formation [36]. However, high temperatures may promote anthocyanin breakdown in strawberries, which is mostly catalyzed by *POD* and laccase (*LAC*) genes [35]. The study conducted by Zhang et al. [35] discovered that the FPKM values of anthocyanin degradation-related genes, such as *POD3*, *POD6*, *POD63*, and laccase (*LAC9*, and *LAC14),* were significantly lower than those of anthocyanin synthesis-related genes. Furthermore, an intracellular laccase is responsible for the degradation of anthocyanin in the pericarp of litchi fruit, induced by epicatechin [38].

While woody plants may not exhibit much conservation, *LAC* genes are likely to have a high degree of it. Moreover, the promoter sequences of VvmiR397a, *VvLAC4*, *VvLAC11*, *VvLAC14*, and *VvLAC17* contain gibberellin-responsive cis-acting elements such as TATC-box, GARE, and P-box, suggesting that these genes might play a role in regulating grape growth and development in response to GA [39].

**Figure 2:**
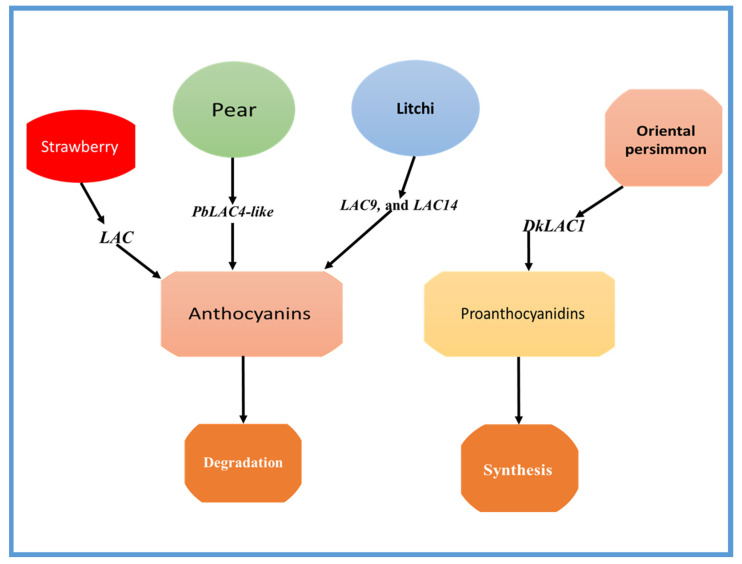
The role of the *LAC* genes in anthocyanin degradation in litchi, pears, and strawberry, and the role of PA biosynthesis in Oriental persimmon (*Diospyros kaki* Thunb.) is shown as an example. *PbLAC4-like* is thought to be connected to the color fading of pears. *PbLAC4-like* plays a role in anthocyanin degradation. *LAC9* and *LAC14* have a role in anthocyanin degradation in litchi fruit. Further, *DkLAC1* plays a role in PA biosynthesis in Oriental persimmon (*D. kaki*).

*PbLAC4*-like is thought to be connected to the color fading of pears. This study found that there was a negative relationship between *PbLAC4*-like expression levels and the amount of anthocyanin present during the fading of pear leaves, petals, and receptacles (Figure 2). Overexpression of *PbLAC4*-like in pear fruitlet peel and enzyme activity tests confirmed its role in anthocyanin degradation [11]. Moreover, the researchers have also investigated the regulator of *PbLAC4*-like and found that PbMYB26 can directly bind with *PbLAC4*-like’s promoter to increase its expression [11].

On the other hand, a gene encoding laccase (*LAC*) has been identified as a possible contributor to the polymerization of proanthocyanidins in the fruit of Oriental persimmon (*Diospyros kaki* Thunb.) [4]. The *DkLAC1* is a laccase found in plants and is closely related to *AtLAC15*, an enzyme known for its involvement in the polymerization of PAs. The expression patterns of genes responsible for PA biosynthesis were studied in three varieties of Oriental persimmons, and it was found that the expression levels of *DkLAC1* in *C-PCNA*-type plants were linked to decreased soluble PA content in the fruit flesh [4]. The role of some important *LAC* genes in fruit coloration is shown in Table 1.

## 4. Molecular Regulation of the *LAC* Gene Family Involved in the Abiotic Stress Response in Fruits

Laccase enzymes, such as OsChI1, have been found to be highly expressed in response to various environmental stresses, including salt, drought, low temperatures, and heavy metal toxicity [12,40]. In fact, overexpression of *OsChI1* and *OsLAC10* in rice has been shown to increase tolerance to salt, drought, and copper toxicity in Arabidopsis [12,13]. Furthermore, upregulation of laccase expression is commonly observed under low-temperature stress, as demonstrated by studies on various *LAC*s in rice, carrot, eggplant, and orange [41,42,43]. However, there are exceptions, such as the downregulation of *SmLAC12* under cold treatment [43] and distinct expression patterns among the 25 *PbLACs* in pear following cold treatment [44]. The contribution of the citrus laccase gene *CsLAC18* to cold tolerance has been identified [45]. The amplification of *CsLAC18* resulted in elevated cold tolerance in tobacco plants, while inhibiting *CsLAC18* via VIGS in *Poncirus trifoliata* diminished its ability to withstand cold stress [45].

## 5. Molecular Regulation of the *LAC* Gene Family Involved in the Protection of Fruits from Diseases

The *LAC* (laccase) gene family encodes oxidoreductases, which play a critical role in the protection of fruits from fungal and bacterial diseases [31]. Laccases catalyze the oxidation of phenolic compounds to quinones, which can immobilize pathogens by cross-linking their cell wall components or by producing toxic compounds. In addition to their antifungal and antibacterial properties, laccases also contribute to the browning of fruits during storage and processing, which can affect their shelf-life and nutritional quality [5,31].

According to recent studies [6,46,47], laccase genes play a vital role in enhancing resistance against pathogen infection. In cotton, *GhLAC1*, *GhLAC4*, and *GhLAC15* are involved in regulating lignin biosynthesis, increasing the lignin content, and enhancing the resistance of cotton to *Verticillium dahliae* infection. Similarly, in Chinese pear (*Pyrus bretschneideri*), *PbrLAC* genes regulate lignin biosynthesis, and their transient silencing can decrease the lignin content and the number of stone cells in fruits [31]. To understand how miR397 regulates lignification through laccases in pear, Yang et al. [5] conducted experiments employing 5′-RNA ligase-mediated-RACE and co-transformation in tobacco to study the effects of PcmiR397 on the expression of *PcLAC*s. Ref. [5] also investigated the expression patterns of PcmiR397 and *PcLAC* target genes in response to pathogens in pear. The silencing of PcmiR397 and overexpression of a single *PcLAC* in pear increased resistance to pathogens through enhanced lignin synthesis [5]. This shows the potential role of *PcmiR397-PcLACs* in providing broad-spectrum resistance to fungal diseases in pear. The molecular regulation of the *LAC* gene family is a complex process that involves multiple layers of signaling and transcriptional control. Understanding these regulatory mechanisms can provide insights into the molecular basis of fruit disease resistance and the development of strategies to enhance fruit quality and shelf-life. The role of some important *LAC* genes in the protection of fruits from diseases is shown in Table 1.

## 6. Conclusions

Laccases are copper-containing oxidases that are widely distributed in the plant kingdom. They play an important role in lignin synthesis, which is essential for plant growth and development. Lignin is a complex polyphenolic polymer that imparts mechanical strength and stability to cell walls. Moreover, it provides barriers to pathogens, insect pests, and environmental stress. Laccases catalyze the polymerization of monolignols into lignins by oxidative coupling reactions, where a radical is generated and coupled with another monolignol to form a dimer. The dimer is then further coupled in vitro to produce higher order polymers.

This article summarizes multiple studies conducted on various fruit-bearing plants, including apples, grapes, and tomatoes, to understand the role of *LAC* genes in fruit development. The studies indicated that the *LAC* gene family is highly conserved in fruit-bearing plants, and their expression is closely associated with fruit development, ripening, and quality attributes. Through gene expression analysis, the researchers identified that *LAC* genes are expressed in various fruit tissues, including the peel, pulp, and seed. The expression levels of *LAC* genes were found to be influenced by external factors and fruit maturity. Furthermore, the study also showed that the manipulation of *LAC* genes can significantly impact fruit quality attributes, including taste, color, and texture [33]. For instance, the overexpression of *LAC* genes in pears led to improved fruit color fading and increased resistance to pathogens. While in the apple, *LAC* like *MdLAC7* has a role in the apple peel browning [34]

During fruit development, the endocarp, which is the innermost layer of the fruit wall, undergoes lignification. This process is regulated by laccases, which are highly expressed in the endocarp cells. Laccase activity is essential for the deposition of lignin in the endocarp cell walls, which makes them resistant to mechanical damage, microbial infection, and water loss. The lignified endocarp also helps in the dispersal of seeds by mechanical forces such as wind, water, or animal gut passage. Therefore, the success of fruit growth and survival is primarily dependent on the lignification of the endocarp, which is mediated by laccases.

Given the importance of laccases in fruit growth, breeders can use this knowledge to select fruit-bearing plants that have enhanced laccase activity. This can be achieved by screening for natural laccase mutants or by using transgenic approaches to overexpress laccase genes. Plants that have higher laccase activity will have better endocarp lignification, which will increase fruit resistance to pathogens and pests, reduce fruit spoilage, and prolong shelf-life. Furthermore, lignified endocarps can also be utilized for the production of bio-based materials such as fibers, biofuels, and bioplastics.

Moreover, the *LAC* (laccase) gene family plays a crucial role in the lignification process during fruit growth. As research on the *LAC* gene family increases, future prospects for their application in fruit breeding and production are expected to grow. Firstly, the identification and isolation of *LAC* genes in different fruit species could help breeders develop new cultivars with improved fruit quality, shelf-life, and disease resistance. For example, in pears, overexpression of *LAC* genes has been shown to improve the fruit’s resistance to fungal pathogens [5]. Secondly, the use of genomic tools could aid in the identification of key regulatory factors that control *LAC* gene expression during fruit growth and development. This information could be utilized to manipulate *LAC* gene expression to influence fruit composition, texture, and nutritional value. Lastly, the application of biotechnology in breeding could help transfer *LAC* genes from one fruit species to another, potentially improving fruit quality, yield, and disease resistance. Thus, the study of the *LAC* gene family in fruits presents exciting prospects for improving fruit production, quality, and nutrition. Future research will likely continue to uncover the potential applications of *LAC* genes in fruit breeding, and their use in fruit production could lead to more sustainable, disease-resistant, and nutrient-rich fruits. In conclusion, laccases are essential enzymes in fruit growth, particularly in endocarp lignification. This process is critical for fruit defense and seed dispersal, making it an important trait for plant breeding. The understanding of laccase biology in fruit development will enable breeders to develop cost-effective, disease-resistant, and nutrient-rich fruit-bearing plants in the future. Further, this review article provides insightful information into the *LAC* gene family’s role in fruit development and highlights the potential of manipulating these genes to improve fruit quality and enhance agricultural productivity.

## Figures and Tables

**Table 1:** Role of the *LAC* genes in fruit quality production.

Species	Genes	Function	Reference
	**Endocarp lignification**		
Peach	*PpLAC7*, *19*, *20*, *21*, *27*, *28*, and *30*	Contribute to lignin synthesis and endocarp hardness in peach fruit.	[27]
	*PpLAC20* and *PpLAC30*	PpMYB63, which is similar to AtMYB58 and AtMYB63, have the ability to activate the promoters of *PpLAC20* and *PpLAC30.*	[27]
Pomegranate	*PgLAC16*	Increased expression of *PgLAC16* may impede the accumulation of storage material during seed development, leading to the prevention of seed hardness in soft-seed pomegranate.	[2]
	*PgLAC37* and *PgLAC50*	Hard-seed pomegranate cultivar exhibited more significant amounts of *PgLAC37* and *PgLAC50.*	[2]
Walnut	*JrLAC12-1*, *JrLAC12-2*, and *JrLAC16*	Play a role in lignin biosynthesis.	[30]
**Flesh lignification**			
Pear	*LAC* genes	The reduction in lignin content and stone cell number in pear fruit was observed when PbrmiR397a was overexpressed and three *LAC* genes were simultaneously silenced.	[8]
	*LAC18*	PbrMYB169 protein was found to activate the promoters of several lignin genes including *LAC18* through binding with AC elements [ACC(T/A)ACC].	[31]
Kiwifruit	*LAC* genes	The expression of *LAC* genes was correlated with the production of lignin, which contributes to the firmness of the fruit.	[32]
**Fruit ripening**			
Oranges	*LAC4*, *17*, *22*	Fruit ripening and quality development.	[33]
**Anthocyanin and PA degradation/ Biosynthesis**			
Apple	*MdLAC7*	Apple peel browning by producing pigments.	[34]
Pear	*PbLAC4-like*	Anthocyanin degradation.	[11]
Strawberry	*LAC9*, and *LAC14*	Anthocyanin degradation.	[35]
Oriental persimmon (*Diospyros kaki* Thunb.)	*DkLAC1*	The *DkLAC1* is a laccase gene found in plants and is closely related to *AtLAC15*, an enzyme known for its involvement in the polymerization of PAs.	[4]
**Protection of fruits from diseases**			
Chinese pear	*PbrLAC*	PcmiR397 and overexpression of a single *PcLAC* in pear increased resistance to pathogens through enhanced lignin synthesis.	[5]

## Data Availability

Not applicable.
